# Who requires dental treatment under general anesthesia due to pain and severe dental anxiety? Findings from panoramic X-ray images and anamnesis

**DOI:** 10.2340/aos.v84.42895

**Published:** 2025-02-04

**Authors:** Vilde Aardal, Caroline Hol, Anne Rønneberg, Sudan Prasad Neupane, Tiril Willumsen

**Affiliations:** a Oral Health Centre of Expertise in Rogaland, Stavanger, Norway; b Institute of Clinical Dentistry, University of Oslo, Oslo, Norway; c National Centre for Suicide Research and Prevention, Institute of Clinical Medicine, University of Oslo, Oslo, Norway

**Keywords:** Dental anxiety, dental status, abuse, general anesthesia, patient profile

## Abstract

**Objective:**

The aims were to describe the dental status and mental and somatic health profile of patients with severe dental anxiety requiring dental treatment under general anesthesia (GA) in Rogaland County, Norway (2018–2021), and to compare patients with and without reported abuse history.

**Material and methods:**

Dental status was assessed by panoramic X-rays. General health variables were collected from patient records. Statistical comparisons of patients with and without abuse experience by tests of association (significance level *p* < 0.05).

**Results:**

38 women and 18 men (mean ± standard deviation [SD]: 37.9 ± 9.2 years) were included; 27 reported abuse experience. Dental assessment showed 4.6 ± 3.8 missing teeth, 4.8 ± 3.0 root remnants, 5.8 ± 3.7 teeth with caries and 2.6 ± 1.9 teeth with apical periodontitis, among patients with ≥1 of the respective findings. 57.1% used analgesics due to dental pain. 55.4% had comorbid psychiatric conditions; 35.7% used psychopharmaceuticals. There were no statistical differences in dental variables but the abuse experience group had higher frequencies of comorbid psychiatric disorders (*p* = 0.01) and mixed somatic conditions (*p* = 0.03).

**Conclusions:**

Patients with severe dental anxiety requiring dental treatment under GA have complex health problems. They need access to treatment under GA, as treatment of serious odontogenic conditions is otherwise unmanageable.

## Introduction

Dental anxiety is a global issue affecting approximately 15% of people worldwide [1] and may be present either in isolation or as part of a multimorbidity [2–4]. While some individuals develop dental anxiety as a result of negative experience with dental treatment [2, 3], others may experience such anxiety due to previous traumatic events. Research on individuals who have experienced sexual abuse has indicated that certain dental treatment situations may evoke feelings reminiscent of prior abuse experiences [5–7]. Individuals with dental anxiety often refrain from seeking necessary dental treatment, and thus, it is associated with deterioration of the dentition and subsequent functional and social consequences [8–13]. For some of these patients with severe dental anxiety, it may be impossible to receive dental treatment without general anesthesia (GA) [14].

The dangers of untreated odontogenic infections pose an additional challenge in patients with severe dental anxiety who avoid treatment. Both in terms of the association with general health outcomes, as well as spread of infections with odontogenic origin resulting in more serious infections such as soft tissue abscesses [15], sinusitis [16, 17] or osteomyelitis [18]. Patients with dental anxiety who avoid dental treatment may instead turn to the use of pain killers (analgesics) for immediate relief [19], the persistent use of which may result in multiple negative consequences. Berggren recommended the consideration of dental treatment under GA in combination with cognitive behavioral therapy as an option for patients with dental anxiety in the following situations (1) if a patient needs treatment due to acute dental pain, (2) if the treatment need is extensive and the likelihood of developing pain in the near future is high, or (3) if the patient has been referred specifically for dental treatment under GA and therefore expects to receive it [20]. Most of the literature about the use of GA in dentistry focuses on fearful children and concludes that dental treatment with GA improves health-related quality of life, experienced improvements in social well-being [21], and also may prevent future dental anxiety [22].

In many countries the availability of GA for dental patients is very low with long waiting lists. Despite the reported positive health effects reported from use of GA, there seems to be scant information regarding oral health needs as well as the mental and general somatic health profile in population with dental anxiety unable to receive dental treatment without GA.

In Norway, a dedicated service called the TADA-service (Torture, Abuse, Dental Anxiety) provides both dental anxiety treatment and dental treatment for individuals with severe dental anxiety. The TADA service facilitates access to GA to patients who have started or completed dental anxiety treatment but are unable to undergo dental procedures while conscious, and up until 2021, the TADA service in Rogaland County also offered dental care to patients awaiting anxiety treatment when necessary. An interdisciplinary TADA team consisting of a licensed psychologist and a dentist determines patient admission into the TADA service as well as the urgency and appropriateness of dental treatment under GA. Clinical experience in the TADA service clearly supports the scarce research on associations between abuse, dental anxiety, and oral health status and diseases in adults [5, 6, 23].

Dental anxiety has been described in literature as a vicious cycle that is self-driven and even self-exacerbating [8, 24–28]. So, the question remains: Who are those who get trapped in the cycle and end up in need of dental treatment under GA? To address healthcare needs, we need to understand their clinical characteristics.

Thus, the primary aim of this study was to describe dental status radiologically in patients with severe dental anxiety who require dental treatment under GA. The secondary aim was to describe the patients’ mental and general somatic health profile. The final aim was to examine possible differences in oral, general somatic, and mental health variables between patients with and without a reported history of abuse.

## Materials and methods

### Study design and population

This study is a retrospective cross-sectional quality assessment. The inclusion criteria were: (1) Enrollment in the TADA service, Rogaland County, Norway, prior to undergoing anxiety treatment, (2) treatment at the Oral Health Centre of Expertise (OHCE) due to pain, (3) inability to receive dental treatment without GA, (4) available panoramic X-rays, and (5) available anamnestic information. Exclusion criteria were: (1) Patients who did not receive treatment under GA. Study information and an opt-out consent form were sent via postal mail to all TADA-patients who were registered at the OHCE between 2018 and 2021. A total of 58 patients were eligible for the study; one refused to participate, and one was excluded due to poor panoramic X-ray image quality.

The study was approved by the Norwegian Agency for Shared Services in Education and Research (Sikt, reference 793691).

### Data collection and variables

Panoramic X-ray imaging (Myray, Cefla Dentale, Italy) was done at the OHCE by trained dental assistants, when not provided in the referral. Due to the potential psychological trauma associated with obtaining intraoral X-rays at the beginning of the TADA treatment, such images are not routinely taken; therefore, only panoramic X-ray images were available for all patients. The images were assessed on a high-resolution monitor (EliteDisplay E241i,HP, USA) which was calibrated for grayscale images [29], in a room with sufficiently low ambient light. The images were displayed using Windows Photos software (Microsoft, USA). The radiological assessment included evaluation of image quality and registration of dental variables. The variables and their definitions are presented in Table 1, and the image quality assessment is presented in Appendix.

**Table 1:** Dental variables assessed by panoramic X-ray imaging.

Variable	Abbreviation	Criteria for registration
Teeth	Teeth	Natural teeth with visible crowns and some occlusal or incisal surface
Root remnants	RR	Teeth without detectable crowns or without any occlusal or incisal crown surface.
Missing teeth	MT	Area in the dental arch with no visible teeth or root remnants. Not including missing premolars with no gap in the dental arch (consistent with previous orthodontic tooth extractions) and not including 3. molar areas.
Dental restorations	DR	Teeth with radiopaque areas consistent with fillings, inlays/onlays or prosthetic crowns
Root-filled teeth	RFT	Teeth or root remnants with radiopaque material consistent with root fillings in one or more root canal, with or without a prosthetic root post
Caries	Caries	Radiolucent lesions in crowns (or cervical root in areas with reduced marginal bone level) consistent with dental caries
Apical radiolucencies	AR	Teeth or root remnants with clearly visible periapical radiolucencies consistent with apical periodontitis

Information was extracted from the anamnesis section of the electronic patient records, and included patient-reported diseases or conditions, medications, and reasons for referral, as well as any record of abuse experience. Abuse is defined by the TADA service as single or repeated incidents of any form of sexual abuse, as well as any variety of physical or mental violence occurring in close relations that the individual either endures or observes [30].

Data from clinical dental examinations were not collected, since not all patients are able to go through an intraoral examination while awake, and examinations under GA do not necessarily include systematic registration of clinical data. All data from the electronic patient records were exported and de-identified by a dental assistant. Only the dental assistant had access to the identification key.

### Radiological assessment

A team of two trained observers performed the radiological assessments; a general dentist (the first author) and an experienced prosthodontic specialist, the latter of whom also conducted the dental treatment under GA. An experienced specialist in maxillofacial radiology (the second author) created an image evaluation template together with the first author before observer calibration. Training was done using 30 anonymized panoramic images obtained from prosthodontic patients who were not part of the study. The two observers evaluated all calibration images individually and then conveyed with the maxillofacial radiologist to reconcile any differences in evaluations. Training was concluded once the observers, aided by the maxillofacial radiologist, reached a consensus on the interpretation of all the variables.

The panoramic images included in the study were initially evaluated separately by the two observers. Following their individual assessments, they convened to compare findings and reach a consensus. If uncertainties arose, the maxillofacial radiologist was consulted to aid in reaching an agreement.

### Assessment of data from the anamnesis in the patient records

Reported medical conditions were interpreted by a medical doctor who then classified those conditions according to the International Statistical Classification of Diseases and Related Health Problems [31]. The identified conditions were organized hierarchically using the Medical Subject Headings thesaurus (National Library of Medicine) at the system level. Medications were pragmatically classified and presented in order of their frequency of ongoing use by the sample.

### Statistics

All statistical analyses were performed using IBM SPSS Statistics version 29. Analyses were run to obtain descriptive statistics (frequencies and means with corresponding standard deviations) for the whole group and for the reported abuse experience (RAE) and no reported abuse experience (NRAE) groups separately. Further, to compare the two groups, Mann-Whitney-U tests were run on linear variables, and Fisher’s exact tests were run on dichotomous variables. Analyses yielding p-values smaller than 0.05 were considered as representing statistically significant differences. Where significant differences were found between groups with and without abuse experience, binary logistic regression was used to control for the effect of gender.

## Results

The included 56 patients had a mean age of 37.9 ± 9.2 years, ranging from 23 to 59 years. In total, 38 of the patients were women. The sample consisted of 27 patients with RAE and 29 without.

### Description of dental status according to radiological findings

**Figure 1:**
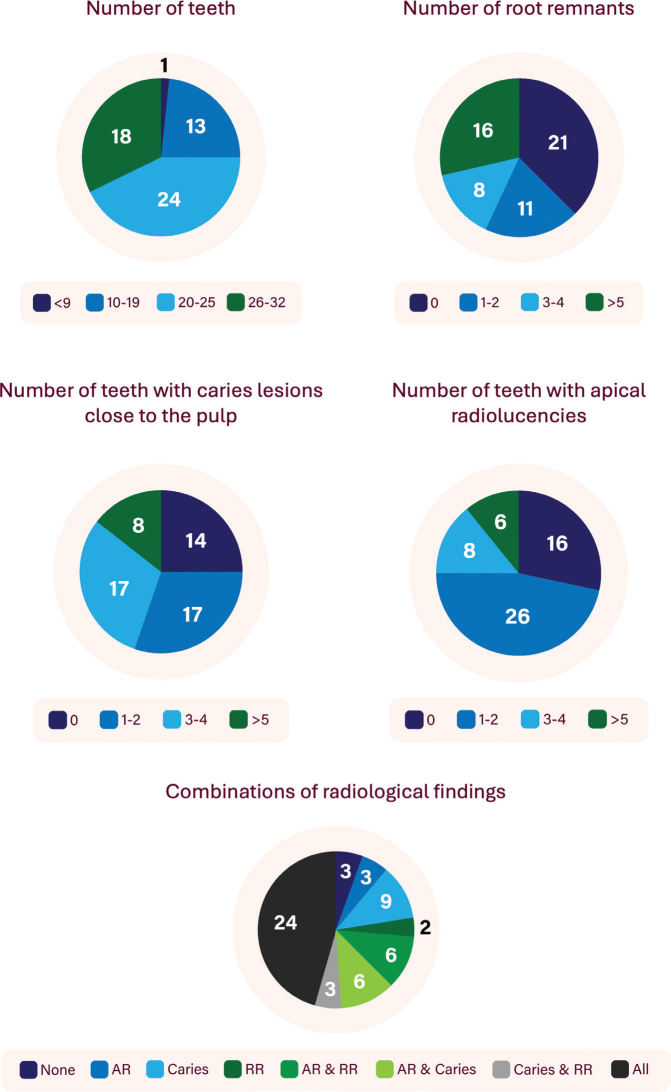
Number of participants with specific radiological findings and their combinations. AR: Apical radiolucencies; RR: Root Remnants.

Table 1 describes each variable studied. Table 2 shows the frequencies of radiological findings for each dental variable and the average number of findings per participant with findings. Figure 1 aggregates the following dental findings in increments and in combinations of findings: number of teeth, number of root remnants, number of teeth with dental caries close to the pulp, and number of apical radiolucencies.

**Table 2:** Findings from panoramic X-rays for the whole sample and comparison of groups.

Variable	Total			RAE	NRAE	*p*

	*N*		Range	*n* = 27	*n* = 29	
Mean no of teeth	56	22.4 ± 5.7	6 – 32	22.3 ± 5.2	22.5 ± 6.3	0.70^C^
Mean no of teeth excluding third molars	56	21.4 ±5.2	6 – 28	21.6 ± 4.8	21.2 ± 5.7	0.90^C^
Missing teeth ≥ 1^B^	56	44 (77.2%)		23 (85.2%)	21 (72.4%)	0.33^D^
Mean no of missing teeth^A^	44	4.6 ± 3.9	1–17	4.4 ± 3.9	4.7 ± 3.9	0.93^C^
Root remnants ≥ 1^B^	56	35 (62.5%)		14 (51.9%)	21 (72.4%)	0.17^D^
Mean no of root remnants^A^	35	4.8 ± 3.0	1–11	4.7 ± 2.3	4.8 ± 3.4	0.83^C^
Teeth with restorations ≥ 1^B^	56	55 (98.2%)		26 (96.3%)	29 (100.0%)	0.48^D^
Mean no of teeth with restorations^A^	55	10.3 ± 5.4	1 – 21	11.3 ± 4.9	9.4 ± 5.8	0.18^C^
Root-filled teeth ≥ 1^B^	56	32 (57.1%)		15 (55.6%)	17 (58.6%)	1.00^D^
Mean no of root-filled teeth^A^	32	3.1 ± 2.3	1 – 10	3.3 ± 2.4	2.8 ± 2.3	0.53^C^
Caries ≥ 1^B^	56	46 (82.1%)		20 (74.1%)	26 (89.7%)	0.17^D^
Mean no of teeth with caries^A^	46	5.8 ± 3.7	1 – 17	6.6 ± 4.0	5.2 ± 3.4	0.38^C^
Apical radiolucencies ≥ 1^B^	56	40 (71.4%)		19 (70.4%)	21 (72.4%)	1.00^D^
Mean no of teeth with apical radiolucencies^A^	40	2.5 ± 1.9	1–10	2.4 ± 2.2	2.6 ± 1.6	0.27^C^

RAE: reported abuse experience; NRAE: no reported abuse experience.

Note.

A Mean ± SD of cases ≥ 1,

B *n* (%),

C Mann–Whitney U test,

D Fisher’s exact test (2-sided).

Although only a single panoramic image was excluded due to poor image quality, all images exhibited at least one variable that could not be assessed for every tooth or root remnant. The areas most affected by suboptimal image quality were from canine to canine in both jaws. Suboptimal image quality most often compromised the assessability of apical radiolucencies and caries lesions. On average, 71% of teeth in each image were assessable for caries, and 55.4% of teeth or root remnants were assessable for apical radiolucencies.

Among patients with fewer than 20 teeth (*n* = 14), 13 (93%) presented with at least one apical radiolucency and 12 (86%) had at least one tooth affected by caries. Patients with 20 or more teeth (*n* = 42) presented less frequently with apical radiolucencies (64%, *p* = 0.47), but almost as frequently with dental caries (81%, *p* = 1.00).

### Description of mental and general somatic health profile

Morbidity was high in the current sample, with 42 (75%) reporting at least one condition. Psychiatric disorders other than severe dental anxiety were reported by 32 (55.4%) patients, with non-specified psychiatric disorder being the most common (21.4%), followed by Post Traumatic Stress Disorder (PTSD), anxiety, and depression. Table 3 further details the anamnestic information reported by the patients. A substantial proportion of the sample reported mixed somatic conditions (35.7%), oral conditions and habits (21.4%) and developmental anomalies or genetic conditions (16.1%). Use of medication was commonplace among the sample, with 62.5% reporting use of at least one class of medication. Psychopharmacological agents (35.7%) and analgesics (33.9%) topped the list (Table 3). Additionally, 57.1% reported taking pain medications due to the dental pain they sought treatment for.

**Table 3:** Anamnestic information including reported conditions and medication use by abuse experience.

Variable	Total^A^	RAE^A^	NRAE^A^	P^B^	Reported conditions (*n*)

	*n* = 56	*n* = 27	*n* = 29		
Oral conditions and habits	12 (21.4%)	8 (29.6%)	4 (13.8%)	0.20	Xerostomia (9), tooth grinding (2)
Developmental anomalies and genetic conditions	9 (16.1%)	7 (25.9%)	2 (6.9%)	0.07	AHDH (4), ADD + ADHD (2), Ehler-Danlos (2), Alport syndrome (1)
Musculoskeletal and connective tissue diseases	5 (8.9%)	3 (11.1%)	2 (6.9%)	0.66	Osteoporosis (2), scoliosis (1), ankylosing spondylitis (1), rheumatism (1)
Cardiovascular conditions	5 (8.9%)	4 (14.8%)	1 (3.4%)	0.19	High blood pressure (3), low blood pressure (1), non-specified CVD (1)
Ear, nose and throat conditions	6 (10.7%)	4 (14.8%)	2 (6.9%)	0.41	Sinus problems (5), tinnitus (1)
Psychiatric conditions other than severe dental anxiety	32 (55.4%)	20 (74.1%)	11 (37.9%)	0.01	Non-specified psychiatric condition (12), PTSD (7), generalized anxiety disorder (2), social anxiety (3), non-specified anxiety (7), panic disorder (1), depression (7), depressive episodes (1), post-partum depression (1), bipolar disorder (1), borderline personality disorder (1), dissociative disorder (1), non-specified eating disorder (1), anorexia (1).
Nervous system conditions	5 (8.9%)	1 (3.7%)	4 (13.8%)	0.35	Multiple sclerosis (1), migraine (3), nerve disease after chemotherapy (1)
Asthma	6 (10.7%)	3 (11.1%)	3 (10.3%)	1.00	
Hearing loss	5 (8.9%)	4 (14.8%)	1 (3.4%)	0.19	
Mixed conditions	20 (35.7%)	14 (51.9%)	6 (20.7%)	0.03	chronic pain (3), immune disorder (3), bleeding disorder (2), diabetes (2), breathlessness/lung disease (2), viral infection (2), GI disease (2), reduced vision (2), physically challenged (1), whiplash injury (1), renal impairment (1), hypothyroidism (1), endometriosis (1)
Analgesic medications	19 (33.9%)	14 (51.9%)	5 (17.2%)	0.01	
Psychopharmacological medications	20 (35.7%)	12 (44.4%)	8 (27.6%)	0.27	
Gastrointestinal medications	9 (16.1%)	5 (18.5%)	4 (13.8%)	0.73	
Allergy and asthma medications	10 (17.9%)	7 (25.9%)	3 (10.3%)	0.17	
Sleeping medications	6 (10.7%)	5 (18.5%)	1 (3.4%)	0.10	
Antidepressant medications	7 (12.5%)	4 (14.8%)	3 (10.3%)	0.70	
Other medications	13 (23.2%)	7 (25.9%)	6 (20.7%)	0.76	

RAE: reported abuse experience; NRAE: no reported abuse experience; PTSD: post traumatic stress disorder; CVD: Cardiovascular disease.

Note.

A . (%),

B Fisher’s exact test (2-sided).

Cells under the category of psychiatric diagnosis and other conditions do not sum up due to more than one condition per category per participant.

### Differences in oral, general somatic, and mental health variables between patients with and without a reported history of abuse

There were no statistically significant differences between the RAE group and the NRAE group related to age (*p* = 0.23) and gender (*p* = 0.40) distribution.

No statistically significant differences were observed between the RAE and NRAE groups on the number of teeth, missing teeth, root remnants, teeth with restorations, root-filled teeth, teeth with caries lesions or apical radiolucencies (Table 2). Compared to the NRAE group, the RAE group reported a significantly higher rate of psychiatric disorders other than severe dental anxiety, mixed conditions, as well as greater use of analgesic medications (Table 3). Binary logistic regression analyses confirmed increased odds of psychiatric disorders, mixed somatic conditions as well as the use of analgesic medication among patients with an abuse experience. These findings withstood adjustment for gender (Table 4).

**Table 4:** Predictive contribution of reported abuse history and gender on reported psychiatric disorders, other disorders, and use of analgesic medications.

Variable	Psychiatric disorders^A^			Mixed somatic conditions^B^			Analgesic medications^C^		
	B	*p*	OR (CI)	B	*p*	OR (CI)	B	*p*	OR (CI)
Abuse	1.50	0.01	4.48 (1.42, 14.16)	1.36	0.03	3.91 (1.19, 12.85)	1.67	0.01	5.32 (1.54, 18.41)
Gender	0.52	0.40	1.68 (0.50, 5.65)	0.83	0.23	2.29 (0.60, 8.77)	-0.21	0.75	0.81 (0.22, 2.97)

OR: odds ratio; CI: confidence interval.

Note. Binary logistic regression including two dummy coded independent variables: Abuse (1 = reported abuse experience, 0 = no reported abuse experience) and Gender (1 = female, 0 = male). Dependent variables for all models were coded as 1 = yes and 0 = no. The models explain 18.4%

A / 17.4%

B / 18.0%

C (Nagelkerke R square) of the variance observed in the dependent variable.

## Discussion

This study found that patients with severe dental anxiety receiving dental treatment under GA presented poor dental status and frequent dental pathology consistent with having dental-related pain. Information extracted from the anamnesis section of the electronic patient records showed high rates of reported co-occurring psychiatric conditions other than severe dental anxiety, and mixed somatic conditions. Patients who reported a history of abuse experience were more likely than those without an abuse experience to have a co-occurring psychiatric or somatic condition and a higher use of analgesic medications.

Two-thirds of the study patients were women, which is unsurprising both because there are more women in the TADA service than men [32, 33], and because literature consistently finds a higher frequency of dental anxiety among women than men [1]. It is unclear whether the higher frequency of women seeking dental care is due to greater prevalence of dental anxiety or simply a greater willingness to seek help.

Despite the relatively young age of patients in the present study, the number of remaining teeth was markedly lower than that reported both in a recent study of 65-year-olds in Oslo, Norway [34], and the average among the adult population in central Norway [35]. Generally, satisfactory chewing functionality is said to be present when an individual has at least 10 occluding tooth pairs (typically anterior teeth and premolars). In this study, the average number of teeth excluding wisdom teeth was 21.5, and as many as 25% had fewer than 20 teeth, which is remarkably few considering the oldest participant not even being 60 years of age. One study found that a decade ago, among Norwegian 30–59-year-olds, 98.7% had 20 or more teeth [36].

In our study, patients were 11 times more likely to have at least one root remnant than in another sample of individuals twice their age from the general population [37], which should be considered an important finding in terms of risks for chronic infections and reduced oral function. Few studies have explored the average number of root remnants among individuals in Norway. However, a study from the 1990s revealed that the average among elderly dentate individuals was 0.17 [38]. Another Norwegian study with clinical data from 2017–2019 reported at least one root remnant in 5.5% of elderly individuals aged 70 or older [37].

The high number of missing teeth and root remnants underscore the necessity of a program facilitating dental rehabilitation treatment for TADA patients.

Even though the current study only included apical radiolucencies that were conspicuously evident and consistent with apical periodontitis, the rate of apical radiolucencies also exceeded that of apical periodontitis recorded in the general population elsewhere, which has been reported to be just over 40% [39, 40]. Suspected but uncertain radiolucencies that would require other imaging techniques to decide presence or absence of pathology were excluded, suggesting that the true frequency of apical periodontitis is underestimated in our study. Considering that odontogenic infections can be the cause of pain and discomfort and may also be associated with serious health risks [41], the high frequency of both root remnants and radiolucencies among study patients calls for attention.

The patients in this study also presented with high frequencies of untreated caries as 4/5 of patients had a minimum of one tooth displaying evident caries lesions on panoramic X-rays. This is notably greater than in the general adult population of central Norway, where 56% of adults have one or more teeth with dentine caries [35]. While it may be expected that individuals requiring treatment under GA would exhibit dental pathology, the extent of treatment needed at a relatively young age is strikingly notable. These findings warrant serious consideration, particularly given that the numbers presented in this study are conservate estimates.

In total, three patients did not present with any obvious radiological finding that would indicate the need for dental treatment, such as caries lesions close to the pulp, apical radiolucencies or residual root remnants (Figure 1). However, all these patients did have deep dental restorations and thus, pulpitis may have been the cause of dental pain.

Concerning general health, this relatively young patient group carried a high disease burden, especially concerning reported co-occurring psychiatric diagnoses. This is largely consistent with literature, suggesting that dental anxiety often coexists with other psychiatric disorders [42]. The direction of this relationship is not fully understood, and it is unclear whether individuals with dental anxiety are prone to developing other psychiatric conditions or vice versa. It makes conceptual sense, however, that some people have an inherent vulnerability to psychiatric conditions such as anxiety disorders, as is also suggested in literature [43]. Furthermore, the high prevalence of reported analgesic use among the sample indicates treatment needs across disease categories.

The findings from the present study suggest that among people with severe dental anxiety, a history of abuse on its own is not related to specific dental conditions; instead, it appears to be more closely related to the prevalence of reported psychiatric and somatic systemic conditions. Approximately half of the study sample reported experiences of abuse, which is only somewhat higher than in the TADA service in general [32, 33]. It is possible that regardless of the varying reasons for developing dental anxiety, the impact of avoidance of oral health care becomes apparent once help is sought and received. Further, the difference in frequency of psychiatric diagnoses may explain the number of patients with a history of abuse in this study. It is reasonable to think that such co-existence of psychiatric conditions may increase the need of GA.

Some limitations of this study need to be discussed. Using panoramic X-ray alone may not be optimal for diagnosing caries and apical periodontitis [44]. There is a general risk of false negative findings for caries, and for apical periodontitis, overlapping ghost images or effects of a narrow focal trough reduce the diagnostic potential in the posterior maxilla as well as in the anterior parts of the dentition. For root-filled teeth there are also risks of false positive findings when only using X-ray imaging since especially smaller apical radiolucencies may represent fibrous healing after a previous infection and not current apical periodontitis. Evident periapical radiolucencies may also represent other, less common diagnoses, that is, cemento-osseous dysplasia or early developmental cysts or tumors. Due to the risk of both false negative and positive findings in panoramic images in general, and especially in images with suboptimal image quality, criteria were developed for both image quality assessment as presented in Appendix, and the registration of teeth and conditions as presented in Table 1. Since care was taken as to not overestimate the presence of pathology, the numbers presented in this paper should be considered as conservative estimates. While unfortunately not available for the present study, systematically collected clinical data should be incorporated in future research to validate the radiological findings. Furthermore, diseases and medications are self-reported by patients and registered in the electronic patient record, which may lead to both over- and underestimation of medical conditions. Clinical experience suggests that some patients in the TADA service who are offered treatment under GA choose to decline due to a fear of losing control. One study on TADA patients suggested that people in the RAE group fear losing control more than people in the NRAE group [32]. This could potentially skew the reported abuse ratio in the current study, and this study could be failing to reflect the actual frequency of reported abuse among patients with severe dental anxiety who need GA. Furthermore, future research should aim to include a larger sample size and offer a more nuanced distinction based on the type and severity of abuse experienced. The present study’s broad definition of abuse and limited sample size may not fully capture the complexity of the issue. Thus, future studies should aim for detailed clinical phenotyping.

So, who are these patients with severe dental anxiety referred for treatment under GA? The findings of this study suggest that they are individuals with few healthy and functional teeth, and a high frequency of chronic dental infections, which untreated have a subsequent high risk of more serious sequelae. Furthermore, many have both dental anxiety and other comorbidities, such as additional psychiatric conditions, which is more prevalent among patients who additionally report a history of abuse. Unless this patient group gets both anxiety treatment and further dental treatment, their already poor dental health will deteriorate even more, and their dental anxiety is likely to persist.

Thus, this study shows that patients with severe dental anxiety who require dental treatment under GA have complex dental and general health problems, with comorbid conditions being more common among those who report a history of abuse. They need access to treatment under GA, as treatment of serious odontogenic conditions is otherwise unmanageable.

## Supplementary Material

Who requires dental treatment under general anesthesia due to pain and severe dental anxiety? Findings from panoramic X-ray images and anamnesis
