# Supplementary material for: Who requires dental treatment under general anesthesia due to pain and severe dental anxiety? Findings from panoramic X-ray images and anamnesis
Source: Acta Odontol Scand. 2025 Feb 4;84:42895. doi: 10.2340/aos.v84.42895 (PMC11865677; doi:10.2340/aos.v84.42895)
Supplement: Who requires dental treatment under general anesthesia due to pain and severe dental anxiety? Findings from panoramic X-ray images and anamnesis [file AOS-84-42895-s1.pdf]

Supplementary material has been published as submitted. It has not been copyedited or typeset by Acta Odontologica Scandinavica.

Appendix

**Image quality panoramic x-rays**		**N=56 (%)**	Additional image quality parameters									
			**Contrast**		**Brightness**		**Tongue against palate**		**Ghost from cervical spine**		**Movement artifacts**	**Nose Piercing**
			Acceptable	Suboptimal	Acceptable	Suboptimal	Yes	No	No or minimal	Suboptimal	Yes	Yes
**Focal trough settings***	All acceptable	39 (69.6)	39	0	39	0	18	21	39	0	0	1
	Most acceptable**	17 (30.4)	15	2	16	1	5	12	16	1	0	0

**Midline, Frankfort horizontal and incisor planes, **Two of tree settings acceptable.*
